# To what extent does severity of loneliness vary among different mental health diagnostic groups: A cross-sectional study

**DOI:** 10.1111/inm.12727

**Published:** 2020-04-30

**Authors:** Khulood Alasmawi, Farhana Mann, Gemma Lewis, Sarah White, Gill Mezey, Brynmor Lloyd-Evans

**Affiliations:** 1 Division of Psychiatry, University College London, London, UK; 2 Population Health Research Institute, St George’s University of London, London, UK

**Keywords:** common mental disorders, discrimination, loneliness, personality disorders, psychosis, stigma

## Abstract

Loneliness is a common and debilitating problem in individuals with mental health disorders. However, our knowledge on severity of loneliness in different mental health diagnostic groups and factors associated with loneliness is poor, thus limiting the ability to target and improve loneliness interventions. The current study investigated the association between diagnoses and loneliness and explored whether psychological and social factors were related to loneliness. This study employed a cross-sectional design using data from a completed study which developed a measure of social inclusion. It included 192 participants from secondary, specialist mental health services with a primary diagnosis of psychotic disorders (n = 106), common mental disorders (n = 49), or personality disorders (n = 37). The study explored differences in loneliness between these broad diagnostic groups, and the relationship to loneliness of: affective symptoms, social isolation, perceived discrimination, and internalized stigma. The study adhered to the STROBE checklist for observational research. People with common mental disorders (MD = 3.94, CI = 2.15 to 5.72, P < 0.001) and people with personality disorders (MD = 4.96, CI = 2.88 to 7.05, P < 0.001) reported higher levels of loneliness compared to people with psychosis. These differences remained significant after adjustment for all psychological and social variables. Perceived discrimination and internalized stigma were also independently associated with loneliness and substantially contributed to a final explanatory model. The severity of loneliness varies between different mental health diagnostic groups. Both people with common mental disorders and personality disorders reported higher levels of loneliness than people with psychosis. Addressing perceived mental health discrimination and stigma may help to reduce loneliness.

## Introduction

Loneliness has become an increasingly studied topic in the general population and recently in mental illnesses. Loneliness is a subjective experience where individuals feel a discrepancy between social relationships that they desire to have and what they actually have (Perlman & Peplau, 1981). It is an unpleasant feeling linked with lack of quality relationships (Perlman & Peplau, 1982). Loneliness is not synonymous with solitude; individuals can experience loneliness when they are alone, but also can experience loneliness while surrounded by others. Within the general population of the United Kingdom, 22% of women and 18% of men reported feelings of loneliness at any given time (Griffin, 2010). There is evidence that loneliness is more prevalent among people with mental illnesses (Borge et *al*., 1999; Lauder *et al*., 2004). Findings suggest that there are many negative effects that loneliness can have on an individual’s physical health, for example, increased cardiovascular morbidity including hypertension, impaired immune function, and premature mortality (Holt-Lunstad *et al*., 2015; Leigh-Hunt *et al*., 2017; Meltzer *et al.,* 2013). Similarly, there can be negative impacts on people’s mental health, such as suicidal ideation and poorer outcomes in depression (Teo *et al*., 2018; Wang *et al*., 2018).

The main mental health diagnostic groups that have often reported loneliness are psychosis, common mental disorders (CMD), and personality disorders (PD) (Ernst & Cacioppo, 1999). CMD are a group of mental health disorders that are more prevalent than other mental health disorders in the community, which include depression, anxiety disorders, obsessive compulsive disorder (OCD), and post-traumatic stress disorder (PTSD). Previous research has found that there were strong associations between loneliness and CMD, especially depression, phobias (including agoraphobia and social phobia), and obsessive-compulsive disorder (Cacioppo *et al.,* 2006; Luanaigh & Lawlor, 2008; Marshall, 1991; Meltzer *et al.,* 2013; Stednitz & Epkins, 2006). Individuals with psychosis have a higher likelihood of experiencing loneliness than individuals without a mental illness, though they had lower odds than people with depression (Meltzer *et al.,* 2013). Furthermore, one study illustrated that individuals with borderline personality disorder experienced severe levels of loneliness in comparison with people with no history of mental illnesses (Liebke *et al.,* 2017). There is, however, a notable lack of other studies that investigate the relationship between PD and loneliness.

## Factors Associated with Loneliness

There is evidence that depressive symptoms and loneliness might have reciprocal influences that negatively impact people’s well-being (Cacioppo *et al.,* 2006). Furthermore, a systematic review concluded that depressive symptoms, state anxiety, and psychotic symptoms were associated with loneliness in people with psychosis (Lim *et al.,* 2018). However, one study found the association between symptoms of depression and loneliness did not persist after accounting for social factors (e.g. social support and social networks) in people with psychosis (Chrostek *et al.,* 2016). The quality of the studies included in the aforementioned systematic review is poor overall, and research into the relationship between psychosis and loneliness is still in its early phases.

Although related to loneliness, social isolation is a different experience. Social isolation is defined as an objective lack of social relationships in different social settings, for example, in individual, group, community, or large social environments (Zavaleta & Samuel, 2014). A recent study found that people with psychosis were more socially isolated; however, they were not as lonely as people with mood disorders (Giacco *et al.,* 2016). Studies including measures of both social isolation and loneliness are rare in both psychosis and mood disorders. Where this has been done, studies have suffered from small sample sizes and lack of validated loneliness measures (Giacco *et al.,* 2016). People with borderline personality disorder who have fewer social relationships reported higher levels of loneliness (Liebke *et al.,* 2017); however, social isolation in itself did not explain the high prevalence of loneliness in people with borderline personality disorder.

A systematic review found that both perceived discrimination and internalized stigma were associated with loneliness in people with psychosis (Lim *et al.,* 2018). However, studies on the relationship between societal perceptions and loneliness are limited, with a lack of studies that investigate discrimination and stigma in CMD and PD.

## Rationale and Current Study Aims

There is some indication from previous literature that there may be differences in severity of loneliness between different diagnostic groups (Giacco *et al.,* 2016), but there are few direct comparisons of patient groups, and reasons for any differences in loneliness between diagnostic groups are poorly understood. Service users’ needs for help with social relationships and loneliness are often under-addressed in mental health services (Pinfold *et al.,* 2015). In the UK context in which this study was conducted, mental health service users are typically supported by a community psychiatric nurse, acting as a ‘care coordinator’. It is acknowledged in policy guidance that addressing mental health service users’ social needs requires an integrated response from the whole health and social care workforce (Allen *et al*., 2016). Understanding the clinical groups most affected by loneliness, and the factors which are associated with and may underpin loneliness in mental health contexts are, therefore, of high relevance for mental health nursing clinicians and researchers.

Our study addresses gaps in knowledge by (a) investigating whether severity of loneliness varies in people using secondary mental health services in different mental health diagnostic groups (psychosis, CMD, and PD), (b) whether social and psychological factors (affective symptoms, social isolation, perceived discrimination, and internalized stigma) might contribute to differences between these diagnostic groups in levels of loneliness, and (c) whether diagnosis, affective symptoms, social isolation, perceived discrimination, and internalized stigma are independently associated with loneliness. Findings may help to understand the drivers of loneliness in a mental health context, inform future intervention development, and identify priority target populations.

## Methods

This study is a secondary analysis based on data collected from The Social Inclusion Questionnaire User Experience (SInQUE) project, a nationally funded research study (Mezey *et al*., 2020). The SInQUE measure (Mezey *et al*., 2013) assesses social inclusion across five domains: social integration; productivity; consumption; access to services; and political engagement. The original study aimed to test the validity, reliability, and acceptability of the measure in people using secondary mental health services. These included individuals with psychosis, CMD, or PD. The London-Bromley Research and Ethics committee approved this study in 2015 (ref IS/LO/1778). We did not use data from the multicomponent ‘SInQUE measure’ in our study to avoid including conceptually overlapping measures in our models of loneliness. The current study will report methods and measures from the original study that is relevant to the current one. This study adheres to the Appendix I for observational research.

### Setting and design

Two-hundred and thirty-eight participants were referred to take part in the SInQUE study. However, 28 did not want to participate, and 18 could not be contacted or did not meet inclusion criteria. A total of 192 participants took part in the current study, including people with primary diagnoses of psychotic disorders (*n* = 106), CMD (*n* = 49), and PD (*n* = 37). Participants were recruited from secondary mental health services in South West London, St Georges NHS Mental Health Trust (SWLSTG), and Camden and Islington (C & I) NHS Foundation Trust. The range of secondary mental health teams involved were community mental health teams, community rehabilitation teams, complex depression and trauma teams, early intervention in psychosis teams, personality disorder services, and forensic outreach teams. The recruitment process occurred from 22 December 2015 to 22 May 2017. The current study used a cross-sectional study design involving questionnaire data.

### Procedure

An information sheet describing the study was given to managers of 39 community mental health teams in the trusts. Clinicians were asked to identify possible participants that met inclusion criteria. Twenty-two mental health teams made referrals to the research team (9 from C&I and 13 from SWLSTG). Interested potential participants then met a researcher who explained the study and answered their questions. Verbal and written consent was given before data collection began. Study measures were completed through participants’ self-report in a structured, face-to-face interview with a research assistant. Participants received 20 British Pounds after each interview as compensation for their time.

### Participants

The inclusion criteria were as follows: adults over 18 years old and a primary diagnosis by The International Classification of Diseases (ICD)-10 from their electronic health records as having either psychosis (including schizophrenia, bipolar affective disorder, or schizoaffective disorder), CMD (including depressive disorders, anxiety disorders, or obsessive compulsive disorder), or PD (including emotionally unstable personality disorder, dependent personality disorder, or unspecified personality disorder). The individuals needed to be under secondary mental health service care, not currently receiving mental health inpatient or crisis care, and must have had at least one previous hospital admission or period of community crisis care. People with depression or anxiety under secondary care are often different than people with depression or anxiety in the general population, who are mostly treated in primary care. The participants were also required to comprehend English.

### Measures

The main exposure variable was diagnosis (psychosis, CMD, or PD). The potential social and psychological variables included affective symptoms, social isolation, perceived discrimination, and internalized stigma. The main outcome variable was loneliness. Interviews consisted of several standardized questionnaires and the collection of socio-demographic variables and clinical history of participants. Participant information consisted of socio-demographic data (age, gender, ethnicity, civil status, education, employment); diagnosis; length of service contact. We have used the Appendix I for our cross-sectional study.

### *UCLA Loneliness Scale (ULS-8)* (Hays & DiMatteo, 1987)

ULS-8 is a self-reported scale that measures subjective feelings of loneliness. It is a revised version of the ULS-20 (included 20 items) and ULS-4 (included 4 items). ULS-8 has eight items that are rated by participants as either O (‘I often feel this way’), S (‘I sometimes feel this way’), R (‘I rarely feel this way’), or N (‘I never feel this way’). The range of total scores on the ULS-8 is 0-32. ULS-8 was previously found to have good validity, reliability, and acceptability (Hays & DiMatteo, 1987).

### *Affective Symptoms: Brief Psychiatric Rating Scale (BPRS)* (Overall & Groham, 1962; Ventura *et al*., 1993)

The BPRS measures psychiatric symptoms like depression, anxiety, hallucinations, or unusual behaviour. The BPRS has a total of 18 items. Items on this scale are rated from 1 (no presence) to 7 (severe). This study used the ‘affective symptom subscale’ (BPRS items 1 to 4) which included items of depression, anxiety, guilt, and somatic symptoms. The affective subscale is rated based on people’s self-report. The range of scores on the affective subscale is from 1 to 28. A rating of 2 or 3 on any individual item would indicate mild concerns but no disruption in functioning. A rating of 4 or 5 would indicate some moderate issues in functioning. A rating of 6 or 7 indicates disruption in functioning and extreme preoccupation with symptoms. The scale has good reliability and good validity (Anderson & Harvey, 1988).

### *Social Isolation: Adapted Social Outcomes Index (SIX)* (Priebke *et al*, 2008)

The SIX scale measures objective social outcomes. The scale includes four questions about employment (0-2); accommodation (0-2); partnership/family support (0-1); and friendship (0-1). SIX scores aim to evaluate the level and variation of social outcomes in individuals with mental illness. The present study combined item 3 (partnership/family support) and item 4 (friendship) to measure ‘social isolation’ (a range of scores from 0-2). Partnership/family support measures participants either living alone (=0) or living with family (=1), and friendship measures either not meeting a friend within the last week (=0) or meeting a friend in the last week (=1). A low score would suggest social isolation (0 would indicate high social isolation, and 2 would indicate low social isolation). This study used data assessed at the time of interview.

### *Discrimination and Stigma Scale (DISC)* (Brohan *et al*., 2013; Thornicroft *et al*., 2009)

DISC is a self-reported scale measuring an individual’s stigma or discrimination experiences related to their mental illness. These include two subscales: unfair treatment from others (items 1 to 21) and stopping self from doing things for fear of discrimination from others (items 22 to 25). DISC includes a variety of domains such as marriage, parenting, housing, leisure, and religious activities. In the present study, unfair treatment was considered ‘perceived discrimination’ and stopping self was considered ‘internalized stigma’. Questions are rated from 0 (not at all) to 3 (a lot). A mean score (range 0-3) is calculated for each subscale by adding each item score and dividing by the number of items in the subscale. DISC mean A (items 1 to 21) was calculated to measure the severity of perceived discrimination experienced. DISC mean B (items 22 to 25) was calculated to measure the severity of internalized stigma experienced. This scale was used to assess participants’ experiences in the past year. DISC has consistently been found to have good validity, reliability, and acceptability (Brohan *et al*., 2013).

Data were collected about the following potential confounding variables known to be associated with loneliness or considered to have clinical relevance: participant socio-demographic characteristics and clinical characteristics. Socio-demographic variables included age, ethnicity, gender, civil status, education, employment status, having children, and living alone or with someone. Clinical characteristics included length of service contact, time since last discharge (months), previous admissions, involuntary admissions, longest admissions, and number of face-to-face contacts with care coordinator or doctor in the last year.

Age and length of service contact were entered as continuous variables. Diagnoses (psychosis as reference group), ethnicity (White as reference group), civil status (single as reference group), and employment (unemployed as reference group) are categorical data, and hence, dummy variables were created when relevant. Ethnicity categories were collapsed into Black, White, Asian, Mixed, and other. Gender was entered as a binary variable (0 = male, 1 = female).

### Data analysis

Statistical analysis was conducted using IBM SPSS version 22 for statistics. Initially, descriptive data were presented for the whole sample and for each diagnostic group. Unadjusted and adjusted regression models were created for each research question. Firstly, a linear regression model was created to examine the relationship between diagnosis and loneliness, and then, all the potential confounding variables were entered (adjusted model). To investigate the secondary hypotheses, multivariable linear regression models were created to see if any relationship between diagnosis and loneliness remained after adjustment for (i) affective symptoms; (ii) social isolation; (iii) perceived discrimination; and (iv) internalized stigma, firstly without adjustment for confounding variables, and then with adjustment for confounding variables in each case. Bivariate tests of association were used to check for collinearity, using established guidance (Dormann *et al.,* 2013) that *r >* 0.7 could be an indicator that variables are closely associated and collinearity may be an issue and it would not sensible to include both variables in a multivariate model. Lastly, multivariable linear regression was used to determine which factors (among all factors: diagnostic groups, social isolation, affective symptoms, discrimination, and stigma) were independently associated with loneliness in a fully adjusted model.

## Results

There were 107 females (56%) and 85 males (44%) in the study, but gender distribution varied in different diagnostic groups (more females in CMD and PD groups). The mean age of participants was 42.4 years (SD = 11.4, 18–74). The majority of participants were of White ethnicities (67%). Most participants were single and never married (76%) and also unemployed (80.7%). The mean length of service contact was 17.46 years. In comparison with other diagnostic groups, participants from the psychosis group mostly comprised of males and Black and Minority Ethnic (BME) groups. In addition, people with PD were more likely to be single. Table 1 illustrates the descriptive data for the sample characteristics.

The mean score for loneliness in the whole sample was 21.65 (SD 5.12), which is indicative of moderate loneliness levels − this is an average rating of three for most items, which corresponds to feeling lonely sometimes (rather than always, or rarely or never). People with PD were the most lonely on average (M = 24.81, SD = 3.73), followed by CMD (M = 23.49, SD = 4.66), and then psychosis who had the lowest mean score (M = 19.69, SD = 4.89). All diagnostic groups had similar mean scores for affective symptoms and social isolation. In comparison with other diagnostic groups, people with PD had the highest mean score for perceived discrimination. Additionally, PD and CMD groups had similar high mean scores for internalized stigma. Table 2 illustrates the descriptive data for loneliness and social and psychological variables.

### Associations between psychiatric diagnosis and loneliness

A linear regression model was created with diagnostic groups as the exposure and loneliness as the outcome.

This regression model accounted for 19% of the variance in loneliness (Table 3 − model 1). Mean loneliness was 3.8 points (CI 2.22 to 5.38) higher for people with CMD compared to psychosis, and 5.1 points (CI 3.38 to 6.87) higher for people with PD compared to psychosis. These differences were statistically significant (*P* < 0.001). An adjusted model (Table 3, model 2) including confounding variables explained 24% of the variance in loneliness but made only minimal changes to the differences in loneliness between diagnostic groups. The adjusted mean loneliness score was 3.9 points (CI 2.15 to 5.72) higher in people with CMD compared to psychosis and 5.0 points (CI 2.88 to 7.05) higher for people with PD compared to psychosis.

### Social and psychological variables in loneliness

We explored if selected social and psychological variables helped contribute to the difference in loneliness between diagnostic groups.

Affective symptoms and social isolation added relatively little difference to our loneliness model and did not substantially change the differences in loneliness between diagnostic groups. After adjustment for affective symptoms (Table 4 − model 2), the mean loneliness score was 3.7 points higher for people with CMD compared to people with psychosis, and 4.7 points higher for people with PD compared to psychosis. After adjusting for social isolation (Table 4 − model 4), the mean loneliness score was 3.9 points higher for people with CMD compared to psychosis and 4.8 points higher for people with PD compared to psychosis.

Perceived discrimination and internalized stigma made a bigger difference in our model of loneliness. After the addition of perceived discrimination into the model, it accounted for 47% of the variance in loneliness (Table 4 − model 6). The mean loneliness score was 3.1 points higher for people with CMD compared to people with psychosis and 2.8 points higher for people with PD compared to people with psychosis. The addition of internalized stigma into the model accounted for 42% of the variance in loneliness (Table 4 − model 8). The mean loneliness score was 2.5 points higher for people with CMD compared to psychosis and 3.4 points higher with people with PD compared to psychosis.

Social isolation and loneliness were found to have a nonsignificant relationship r(190) = —0.11, *P* = 0.14. However, affective symptoms and loneliness were found to have a weak positive correlation *r* (190) = 0.20, *P* = 0.01. Similarly, the link between discrimination and loneliness (r(190) = 0.50, *P* < 0.001.) and between stigma and loneliness were both found to have a moderate positive correlation (r(190) = 0.50, *P* < 0.001.).

Finally, multivariable linear regression was conducted to investigate which factors (affective symptoms, social isolation, perceived discrimination, and stigma) were independently associated with loneliness, after accounting for potential confounders. This regression model accounted for 54% of the variance in loneliness (Table 5 − model 1). Perceived discrimination and internalized stigma remained independently associated with loneliness. For every 1-point increase in perceived discrimination, we expect loneliness to increase by 3.72 points (95% CI 2.47 to 4.98, *P* < 0.001). For every 1-point increase in internalized stigma, we expect loneliness to increase by 1.69 points (95% CI 0.94 to 2.44, *P* < 0.001). Diagnosis still remained independently associated with loneliness in this final model. The adjusted mean loneliness score was 2.4 points higher for people with CMD compared to psychosis and 2.3 points higher for people with PD compared to psychosis.

## Discussion

There was statistical evidence of significant differences found in severity of loneliness between people with CMD and psychosis and between people with PD and psychosis. Given the standard deviation across the whole sample on the ULS-8 loneliness measure of 5.12 points, the unadjusted mean differences in ULS-8 score between the psychosis group and the CMD group (3.80) and PD group (5.12 points) constitute moderate and large effects, respectively (Cohen, 1998). Even our fully adjusted model still indicated a small independent effect on loneliness for diagnosis, unexplained by measured clinical, social or demographic factors.

Our study thus corroborated previous research in finding lower rates of loneliness for individuals with psychosis than the other diagnostic groups (Giacco *et al*., 2016), but with a larger and more clinically varied sample of mental health service users.

The differences in loneliness between diagnostic groups were partially related to the higher rates of perceived discrimination and internalized stigma found in individuals with CMD and PD. However, the differences in loneliness scores between diagnostic groups were not entirely linked to the social and psychological factors. Despite having a good model of loneliness in the final fully adjusted model (*R*^2^ of 54%), unexplained differences in loneliness scores between diagnostic groups remained.

In our final multivariable model, affective symptoms and social isolation were not independently associated with loneliness. Previous research indicated that discrimination and stigma are related to loneliness for individuals with psychosis (Lim *et al*., 2018); our study suggests they are also important for other diagnostic groups.

## Strengths and Limitations

This was the first study to investigate and compare loneliness in CMD, psychosis, and PD using a large sample of people all recruited from secondary mental health services. We were able to explore the relationship of psychological and social factors to loneliness and account for potential important confounding factors.

The main limitation was the cross-sectional study design which does not permit causal inferences to be made. It is difficult to know if the factors are predictors of loneliness or vice versa. It is also difficult to conclude if the social and psychological variables are independent variables that cause loneliness because they were investigated at the same time the exposure and outcome were measured. Another limitation is that our study was conducted in only two areas of London: results may not be generalizable to other areas. Additionally, despite the excellent participant recruitment rate, there may have been an element of volunteer bias because participants who chose to participate in the study may have been different from those who did not want to. Further limitations may include our use of broad mental health diagnostic groups, instead of specific mental illnesses: there may have been differences in indicators of social connectedness within disorders in the groups. Our broad diagnostic groups also did not allow us to explore how comorbidities of the mental health disorders may impact how a person might experience loneliness and may have masked differences in levels of loneliness between people with different diagnoses, within the same broad diagnostic grouping. Our three broad categories, however, have both a research relevance and clinical validity. They allow us to investigate whether a previous research finding (Giacco *et al*., 2016) can be replicated: that people with psychosis are less lonely than those with CMD. They also reflect how secondary mental health services are organized in many areas of the UK, including our study sites: which group someone belongs to determine what services they are offered. In addition, social isolation was measured using two items that may have not been able to capture the complexity of social integration and networks. Another possible limitation is uncertainty of the ULS-8 scale’s ability to detect feelings of loneliness in people with psychosis, although the measure does have some evidence of good criterion validity in a mixed sample of mental health service users including people with psychosis (Wang, 2018).

## Interpretation and Comparison with The Literature

In line with previous literature, people with CMD reported higher levels of loneliness than people with psychosis (Giacco *et al*., 2016). The experience of loneliness in people with CMD may link to reduced enjoyment of social relationships even when they have relationships (Heinrich & Gullone, 2006). Similar to recent findings, we found that people with psychosis had the highest social isolation scores (Giacco *et al*., 2016), but overall social isolation did not differ greatly between groups in our study. However, social isolation may not be perceived by people with psychosis as loneliness because it might be seen as a mechanism for coping with perceived stress (Perivoliotis & Cather, 2009). Levels of perceived discrimination and internalized stigma were higher in the nonpsychosis groups. Previous qualitative studies have also found that people with psychosis experience stigma, but people with CMD or PD reported experiencing it more even if they did not experience any direct discrimination (Dinos *et al*., 2004).

Contrary to previous studies, we found that affective symptoms were not closely related to loneliness (Cacioppo *et al*., 2006; Lim *et al*., 2018; Wang *et al*., 2018): affect appears to drive loneliness less in our study population of secondary mental health service users than for people in primary mental health care or the general population.

Perceived discrimination and internalized stigma seem to be important factors related to loneliness in mental illnesses. This is fitting with theoretical models of loneliness which suggest that hypervigilant or overactive perception of social threat might drive loneliness, such as the ‘reaffiliation motive’ theory (Qualter *et al*., 2015). The theory implies that individuals with mental illnesses perceive that others are against them (whether based in their social world or driven by an inaccurate perception of threat), which could be driving their high levels of loneliness.

## Implications and Future Research

We found that higher levels of loneliness were reported in people with CMD and people with PD. This might mean that people with CMD and PD are priority populations for support from nurses and other mental health staff, for whom loneliness prevention strategies and targeted interventions are needed. Social isolation contributed little to people’s level of loneliness, but perceived discrimination and internalized stigma were important factors that contributed to loneliness. This might suggest that the reality of discrimination and stigma might make individuals feel like ‘outsiders’ and experience severe feelings of loneliness, even if they maintain social relationships. This may have implications for the development and improvement of interventions to decrease levels of loneliness, which consequently might also improve mental health outcomes. It might be important to target self-stigmatization and perceived discrimination and how these factors could affect people’s behaviour and experiences of social contact. Our results also support the potential value of public health anti-stigma and discrimination for mental health campaigns, for example ‘Time to Change’. Our study also indicates approaches to addressing loneliness which might be ineffective. Social isolation was not related to loneliness in our final model and has been found to be only moderately correlated with and loneliness in previous studies, and thus, interventions that aim to increase social contacts alone may not be as effective (Mann *et al*., 2017). Similarly, affective symptoms may not be as important to target in interventions for loneliness in this context. The diagnostic groups remained independently associated with loneliness in a fully adjusted model, which suggested that different mental health diagnostic groups may experience loneliness differently, and may need correspondingly tailored interventions.

It is important for future research to account for comorbidity of the mental health disorders. It is also necessary to investigate loneliness in the three diagnostic groups with a longitudinal study design. This would help us understand the temporality of loneliness and how it develops and changes over time. It would also allow us to make casual inferences and have better understanding of the direction of causation with potential explanatory factors. Additionally, it would be interesting to look at the dynamics between different psychological and social factors with loneliness over time (e.g. perceived discrimination and loneliness) and the interaction of the factors (e.g. perceived discrimination with internalized stigma). More studies that investigate levels of discrimination and stigma in nonpsychosis groups would be helpful in corroborating our results. Additionally, semi-structured interviews with people from different diagnostic groups could help us gain deeper insight into experiences of discrimination and stigma. Further research into creating a loneliness scale that is developed in a population of severe mental illness could be important because the ULS-8 scale was developed in a general population sample and thus may not be ideal or sensitive enough to measure loneliness in severe mental illnesses. Additionally, using samples from various mental health trusts across the UK and beyond would be ideal for findings to be applicable to a wider population.

## Conclusions

The severity of loneliness varies between different mental health diagnostic groups. People with PD and CMD both reportedly experience higher levels of loneliness in comparison with psychosis. Although perceived discrimination and internalized stigma explain some of the differences in loneliness between diagnostic groups, our research implies that loneliness might still be experienced differently in different clinical groups, who could need specific loneliness interventions.

## Relevance for Clinical Practice

Loneliness is an important determinant of mental and physical health and is often under-addressed in mental health services. Our study shows that, across a range of diagnostic groups, people using secondary mental health services often experience high levels of loneliness. However, the extent and experience of loneliness may be different for people with psychosis, compared to those with CMD or PD: tailored interventions for each group may be needed. Addressing societal discrimination and self-stigma are promising ways to help reduce loneliness in mental health. Nurses, as the professional group most commonly supporting mental health service users in secondary care, have a key role to play in helping reduce loneliness and support recovery.

## Supplementary Material

Appendix

## Figures and Tables

**Table 1:** Descriptive data for sample characteristics across diagnostic groups

	Psychosis		Common Mental Disorders		Personality Disorders		Whole Sample	
N	106		49		37		192	
Socio-demographic characteristics								
Age: mean (SD) range	43.7 (10.9) 23–74		45.1 (11.6) 18–71		35.1 (10.0) 19–52		42.4 (11.4) 18–74	
Gender (n/%)								
Male	60	56.6	16	32.7	9	24.3	85	44.3
Female	46	43.4	33	67.3	28	75.7	107	55.7
Ethnicity (*n*/%)								
White	60	56.6	39	79.5	30	81.1	129	67.2
Black	31	29.3	5	10.0	1	2.7	37	19.3
Mixed race	12	11.4	1	2.0	3	8.1	16	8.3
Asian	2	1.9	4	8.0	0	0.0	6	3.1
Other	1	0.9	0	0.0	3	8.1	4	2.1
Marital status (*n*/%)								
Single	82	77.4	31	63.3	33	89.2	146	76.0
Divorced/separated	13	12.3	11	22.4	3	8.1	27	14.1
Widow/er	2	1.9	0	0.0	0	0.0	2	1.0
Married	8	7.5	7	14.3	1	2.7	16	8.3
Highest level of education reached (*n*/%)								
No qualification	33	31.1	7	14.3	6	16.2	46	24.0
GCSE (or equivalent)	27	25.5	15	30.6	16	43.2	58	30.2
A Level	24	22.6	13	26.5	9	24.3	46	24.0
University degree (or equivalent)	21	19.8	13	26.5	6	16.2	40	20.8
Employment status (*n*/%)								
Paid employment	9	8.5	6	12.2	8	21.6	23	12.0
Sheltered employment	2	1.9	0	0.0	0	0.0	2	1.0
Training/education	2	1.9	1	2.0	2	5.4	5	2.6
Unemployed	88	83.0	40	81.6	27	73.0	155	80.7
Retired	4	3.8	2	4.1	0	0.0	6	3.1
Other	1	1.0	0	0.0	0	0.0	1	0.5
Clinical history								
Length of service contact (years) Range	19.4 (10.7) 1–53		15.5 (11.2) 1–45		14.4 (8.3) 2–38		17.5 (10.6) 1–53	

**Table 2:** Mean scores of loneliness (ULS-8) and social and psychological variables across diagnostic groups

	Psychosis	Common mental disorders	Personality disorders	Whole sample
Loneliness				
Mean	19.69	23.49	24.81	21.65
*N*	106	49	37	192
SD	4.89	4.66	3.73	5.12
Affective symptoms				
Mean	7.10	7.88	7.70	7.41
*N*	105	49	37	191
SD	2.47	2.03	2.07	2.30
Social Isolation†				
Mean	0.89	0.92	0.97	0.91
*N*	106	49	37	192
SD	0.62	0.73	0.80	0.68
Perceived discrimination🟒				
Mean	0.49	0.59	0.95	0.61
N	106	49	37	192
SD	0.52	0.44	0.54	0.53
Internalized stigma🟒				
Mean	0.76	1.22	1.48	1.02
*N*	106	49	37	192
SD	0.79	0.91	0.83	0.88

† Low mean score indicates high social isolation.

🟒 There was a significant difference between diagnostic groups in univariate testing *P* < 0.001.

**Table 3:** Multivariable linear regression models of associations between diagnosis and loneliness (ULS-8)

Variable Psychosis	Mean difference (95% CI) Reference	*p*-value
Model 1.		
Common mental disorders	3.80 (2.22 to 5.38)	<0.001
Personality disorders	5.12 (3.38 to 6.87)	<0.001 R^2^ = 0.19
Model 2.		
Common mental disorders	3.94 (2.15 to 5.72)	<0.001
Personality disorders	4.96 (2.88 to 7.05)	<0.001 *R*^2^ = 0.24

Outcome is loneliness.

Model 1 is unadjusted, Model 2 is adjusted for potential confounders (age, gender, ethnicity, civil status, employment, length of service contact).

**Table 4:** Multivariable linear regression models of relationships between diagnostic groups and loneliness, adjusting for social and psychological variables

Variable Psychosis	Mean difference (95% CI) Reference	P-value
Model 1.		
Common mental disorders	3.57 (1.97 to 5.15)	<0.001
Personality disorders	4.94 (3.19 to 6.69)	<0.001 *R*^2^ = 0.21
Model 2.		
Common mental disorders	3.66 (1.87 to 5.46)	<0.001
Personality disorders	4.72 (2.62 to 6.81)	<0.001 *R*^2^ = 0.26
Model 3.		
Common mental disorders	3.83 (2.26 to 5.40)	<0.001
Personality disorders	5.20 (3.47 to 6.94)	<0.001 *R*^2^ = 0.21
Model 4.		
Common mental disorders	3.92 (2.14 to 5.69)	<0.001
Personality disorders	4.83 (2.75 to 6.91)	<0.001
Model 5.		
Common mental disorders	3.28 (1.94 to 4.61)	<0.001
Personality disorders	2.81 (1.25 to 4.36)	<0.001 *R*^2^ = 0.43
Model 6.		
Common mental disorders	3.16 (1.66 to 4.65)	<0.001
Personality disorders	2.78 (0.98 to 4.58)	0.003 *R*^2^ = 0.47
Model 7.		
Common mental disorders	2.57 (1.14 to 3.99)	<0.001
Personality disorders	3.20 (1.58 to 4.81)	<0.001 *R*^2^ = 0.38
Model 8.		
Common mental disorders	2.47 (0.86 to 4.98)	0.003
Personality disorders	3.39 (1.52 to 5.26)	<0.001 *R*^2^ = 0.42

Outcome is loneliness.

Model 1 is adjusted for affective symptoms, Model 2 is adjusted for affective symptoms and potential confounders, Model 3 is adjusted for social isolation, Model 4 is adjusted for social isolation and potential confounders, Model 5 is adjusted for perceived discrimination, Model 6 is adjusted for perceived discrimination and potential confounders, Model 7 is adjusted for internalized stigma, and Model 8 is adjusted for internalized stigma and potential con-founders.

**Table 5:** Final multivariable linear regression models of diagnosis, and social and psychological variables in loneliness (ULS-8)

Variable	Mean difference (95% CI)	*P*-value
Model 1.		
Diagnosis - CMD^†^	2.37 (1.00 to 3.84)	0.002
Diagnosis - PD^†^	2.25 (0.52 to 3.98)	0.011
Affective symptoms:	0.12 (-0.13 to 0.37)	0.34
Social isolation:	-0.75 (-1.65 to 0.15)	0.10
Perceived discrimination:	3.72 (2.47 to 4.98)	<0.001
Internalized stigma:	1.69 (0.94 to 2.44)	<0.001 *R*^2^ = 0.54

Outcome is loneliness.

Model 1 is adjusted for affective symptoms, social isolation, perceived discrimination, internalized stigma, and potential confounders

† Reference group is psychosis.
