# Supplementary material for: To what extent does severity of loneliness vary among different mental health diagnostic groups: A cross-sectional study
Source: Int J Ment Health Nurs. Author manuscript; Available in PMC 2024 Dec 8. (PMC7616998; doi:10.1111/inm.12727)
Supplement: Appendix [file EMS181453-supplement-Appendix.pdf]

14470349, 2020, 5, Downloaded from https://onlinelibrary.wiley.com/doi/10.1111/inm.12727 by Test, Wiley Online Library on [19/07/2023]. See the Terms and Conditions (https://onlinelibrary.wiley.com/terms-and-conditions) on Wiley Online Library for rules of use; OA articles are governed by the applicable Creative Commons License

APPENDIX I:

Strobe statement

Checklist of items that should be included in reports of observational studies

Section/ Topic	Item No	Recommendation	Where reported
Title and abstract	1	(a) Indicate the study's design with a commonly used term in the title or the abstract	Title page
		(b) Provide in the abstract an informative and balanced summary of what was done and what was found	Abstract section
Introduction			
Background/ rationale	2	Explain the scientific background and rationale for the investigation being reported	Introduction section, 'Factors Associated with Loneliness' subsection and 'Rationale and Current Study'paragraph 1
Objectives	3	State specific objectives, including any prespecified hypotheses	Introduction section, 'Rationale and Current Study' and 'Hypotheses'subsection
Methods			
Study design	4	Present key elements of study design early in the paper	Methods; 'Setting and Design'subsection
Setting	5	Describe the setting, locations, and relevant dates, including periods of recruitment, exposure, follow-up, and data collection	Methods; 'Setting and Design' and 'Procedure'subsection
Participants	6	(a) *Cohort study*—Give the eligibility criteria, and the sources and methods of selection of participants. Describe methods of follow-up *Case-control study*—Give the eligibility criteria, and the sources and methods of case ascertainment and control selection. Give the rationale for the choice of cases and controls *Cross-sectional study*—Give the eligibility criteria, and the sources and methods of selection of participants (b) *Cohort study*—For matched studies, give matching criteria and number of exposed and unexposed*Case-control study*—For matched studies, give matching criteria and the number of controls per case	Methods; 'Participants' subsection
Variables	7	Clearly define all outcomes, exposures, predictors, potential confounders, and effect modifiers. Give diagnostic criteria, if applicable	Methods; 'Measures' paragraph 1
Data sources/ measurement	8*	For each variable of interest, give sources of data and details of methods of assessment (measurement). Describe comparability of assessment methods if there is more than one group	Methods; 'Measures' paragraphs 2-5
Bias	9	Describe any efforts to address potential sources of bias	Methods; 'Measures' paragraph 6
Study size	10	Explain how the study size was arrived at	Methods; 'Setting and Design' and 'Procedure' subsection
Quantitative variables	11	Explain how quantitative variables were handled in the analyses. If applicable, describe which groupings were chosen and why	Methods;'Measures' paragraph 7

(*Continued*)

© 2020 Australian College of Mental Health Nurses Inc.

I (Continued)

Section/ Topic	Item No	Recommendation	Where reported
Statistical methods	12	(a) Describe all statistical methods, including those used to control for confounding	Methods; 'Data Analysis' subsection
		(b) Describe any methods used to examine subgroups and interactions	N/A
		(c) Explain how missing data were addressed	N/A
		(d) *Cohort study*—If applicable, explain how loss to follow-up was addressed	N/A
		Case-control study—If applicable, explain how matching of cases and controls was addressed	
		Cross-sectional study—If applicable, describe analytical methods taking account of sampling strategy	
		(e) Describe any sensitivity analyses	N/A

Section/Topic	Item No	Recommendation	Where reported
Results			
Participants	13*	(a) Report numbers of individuals at each stage of study—eg numbers potentially eligible, examined for eligibility, confirmed eligible, included in the study, completing follow-up, and analysed	Methods; 'Setting and Design' subsection
		(b) Give reasons for non-participation at each stage	
		(c) Consider use of a flow diagram	N/A
Descriptive data	14*	(a) Give characteristics of study participants (eg demographic, clinical, social) and information on exposures and potential confounders	Results paragraph 1 and 2
		(b) Indicate number of participants with missing data for each variable of interest	
		(c) *Cohort study*—Summarise follow-up time (eg, average and total amount)	
Outcome data	15*	*Cohort study*—Report numbers of outcome events or summary measures over time	
		Case-control study—Report numbers in each exposure category, or summary measures of exposure	
		Cross-sectional study—Report numbers of outcome events or summary measures	Results paragraph 2
Main results	16	(a) Give unadjusted estimates and, if applicable, confounder-adjusted estimates and their precision (eg, 95% confidence interval). Make clear which confounders were adjusted for and why they were included	Results; 'Associations Between Psychiatric Diagnosis and Loneliness' and 'Social and Psychological Variables' subsections
		(b) Report category boundaries when continuous variables were categorized	
		(c) If relevant, consider translating estimates of relative risk into absolute risk for a meaningful time period	N/A
Other analyses	17	Report other analyses done—eg analyses of subgroups and interactions, and sensitivity analyses	N/A
Discussion			
Key results	18	Summarise key results with reference to study objectives	Discussion paragraphs 1-4
Limitations	19	Discuss limitations of the study, taking into account sources of potential bias or imprecision. Discuss both direction and magnitude of any potential bias	Discussion; 'Strengths and Limitations'subsection

(*Continued*)

© 2020 Australian College of Mental Health Nurses Inc.

14470349, 2020, 5, Downloaded from https://onlinelibrary.wiley.com/doi/10.1111/inm.12727 by Test, Wiley Online Library on [19/07/2023]. See the Terms and Conditions (https://onlinelibrary.wiley.com/terms-and-conditions) on Wiley Online Library for rules of use; OA articles are governed by the applicable Creative Commons License

I (Continued)

Section/Topic	Item No	Recommendation	Where reported
Interpretation	20	Give a cautious overall interpretation of results considering objectives, limitations, multiplicity of analyses, results from similar studies, and other relevant evidence	Discussion;'Interpretation and Comparison with the literature' subsection
Generalisability	21	Discuss the generalisability (external validity) of the study results	Discussion; 'Implications and Future Research' paragraph 1 and 'Relevance for Clinical Practice' section
Other Information			
Funding	22	Give the source of funding and the role of the funders for the present study and, if applicable, for the original study on which the present article is based	Title page

*Give information separately for cases and controls in case-control studies and, if applicable, for exposed and unexposed groups in cohort and cross-sectional studies.

Note: An Explanation and Elaboration article discusses each checklist item and gives methodological background and published examples of transparent reporting. The STROBE checklist is best used in conjunction with this article (freely available on the Web sites of PLoS Medicine at http://www.plosmedicine.org/, Annals of Internal Medicine at http://www.annals.org/, and Epidemiology at http://www.epidem.com/). Information on the STROBE Initiative is available at www.strobe-statement.org.

The numbers of different diagnoses in each group are:

Psychosis:

Bipolar affective: 13
Schizophrenia: 64
Schizoaffective: 20
First episode/ psychosis NOS: 9

CMD:

Depression: 26
OCD:10
Mixed depression and anxiety: 5
GAD:1
PTSD: 3
Agoraphobia:2
Adjustment disorder: 2

PD:

Emotionally unstable personality disorder: 26
Personality disorder: 9
Dependent PD: 1
Dissociative disorder 1:

© 2020 Australian College of Mental Health Nurses Inc.

14470349, 2020, 5, Downloaded from https://onlinelibrary.wiley.com/doi/10.1111/inm.12727 by Test, Wiley Online Library on [19/07/2023]. See the Terms and Conditions (https://onlinelibrary.wiley.com/terms-and-conditions) on Wiley Online Library for rules of use; OA articles are governed by the applicable Creative Commons License
